# Layout of Detection Array Based on Multi-Strategy Fusion Improved Adaptive Mayfly Algorithm in Bearing-Only Sensor Network

**DOI:** 10.3390/s24082415

**Published:** 2024-04-10

**Authors:** Zhan Chen, Yangwang Fang, Ruitao Zhang, Wenxing Fu

**Affiliations:** Unmanned System Research Institute, Northwestern Polytechnical University, Xi’an 710072, China; chenzhan@mail.nwpu.edu.cn (Z.C.); ruitaozhang@mail.nwpu.edu.cn (R.Z.); wenxingfu@nwpu.edu.cn (W.F.)

**Keywords:** bearing-only sensor network, state estimation, DCIF algorithm, cooperative consistency theory

## Abstract

The various applications of bearing-only sensor networks for detection and localization are becoming increasingly widespread and important. The array layout of the bearing-only sensor network seriously impacts the detection performance. This paper proposes a multi-strategy fusion improved adaptive mayfly algorithm (MIAMA) in a bearing-only sensor network to perform layout planning on the geometric configuration of the optimal detection. Firstly, the system model of a bearing-only sensor network was constructed, and the observability of the system was analyzed based on the Cramer–Rao Lower Bound and Fisher Information Matrix. Then, in view of the limitations of the traditional mayfly algorithm, which has a single initial population and no adaptability and poor global search capabilities, multi-strategy fusion improvements were carried out by introducing Tent chaos mapping, the adaptive inertia weight factor, and Random Opposition-based Learning. Finally, three simulation experiments were conducted. Through comparison with the Particle Swarm Optimization (PSO) algorithm, Mayfly Algorithm (MA), and Genetic Algorithm (GA), the effectiveness and superiority of the proposed MIAMA were validated.

## 1. Introduction

With the development of wireless sensor networks, the technology for target tracking and positioning has become increasingly prominent. Sensors can be categorized according to their operational principles into active and passive modes. Compared with the active mode, passive tracking does not require actively transmitting signals, but it locates by receiving signals from the target itself or signals from the external environment [1]. In this process, the low signal-to-noise ratio makes it difficult to distinguish signals and noise, thereby greatly reducing the positioning and tracking performance of the system, and thus, it exhibits highly nonlinear behavior and weak observability. This problem widely exists in the fields of military confrontation, unmanned navigation, and mobile robot positioning. Especially in terms of military confrontation, during the tracking and positioning process, maneuvering targets can significantly shorten the detection distance of active sensors through design modifications, absorbing materials, and interference suppression technology, and lead to a sharp decline in detection accuracy. On the contrary, bearing-only sensors that only rely on target radiation characteristics have become an indispensable tool in the modern anti-stealth field because of their long, effective range and strong concealment capabilities.

Passive detection with bearing-only sensors mainly refers to using angle measurements containing noise to acquire a projection or approximation of the present condition of the target, which has the characteristics of nonlinearity and weak observability. Nonlinear problems of the system are usually solved through various filtering methods. To address the issue of limited observability in the system, the multi-platform collaborative detection method is usually used to solve it [2]. The precision of positioning for the collaborative target is influenced not only by the inherent errors in position and angle measurements of the sensor, but is primarily impacted by the geometric arrangement between the sensor and the target. This type of target localization error, originating from the position errors and measurement errors of the bearing-only sensors and propagating through the relative geometric relationship, is called the geometric dilution of positioning (GDOP) [3,4]. Therefore, researching how to plan a rational sensor network layout to enhance the accuracy of system state estimation has significant academic significance and indispensable practical engineering value. This is particularly true for bearing-only sensor networks, which feature various positioning mechanisms, more complex system models, and limited existing research. Thorough investigation in this domain can offer essential support and technical momentum for the advancement of diverse application areas, including, but not limited to, stealth target tracking, navigation state estimation, and mobile robot positioning.

The detection accuracy of the system is usually described by the Circular Error Probable (CEP) [5,6], GDOP, and Cramer–Rao Lower Bound (CRLB) [7,8,9]. The determinant of the Fisher Information Matrix (FIM) is used to represent the observability measurement of the system, thereby providing a usable optimization index for the detection system [10,11,12]. Further research found that CRLB is equal to the inverse of FIM [13,14], which unified the positioning accuracy index and the observability index.

Consider that the efficacy of passive localization relies on the relative geometry between the sensor and the target [15]. Optimal observation geometry pertains to the relative positioning of the passive sensor and the target, aiming to maximize positioning accuracy [16]. In [17], the optimal observation geometry was configured based on the determinant that maximizes FIM, which can be applied to the deployment of passive sensors. In [18], the impact of geometric structure on multi-sensors in array fusion performance was explained. In [19], a distributed array configuration control scheme was proposed based on bearing-only sensor detection. In [20], a deep learning framework for the passive sensor detection process was also proposed; however, this method relied on datasets and had limited real-time performance.

It is evident that there has been limited research in the field of sensor layout optimization theory, specifically for the bearing-only sensor network. Furthermore, the design of algorithmic performance metrics is often exceedingly complex, making it challenging to meet real-time requirements and ensuring detection accuracy for practical problems. Therefore, the optimal detection array layout for a bearing-only sensor network, which is the central focus of this paper, remains a highly necessary and significant issue.

The main innovative contributions are summarized as follows:

1. The first aspect is to construct an observation model based on a bearing-only sensor network, and the optimal detection array of the system is theoretically analyzed based on CRLB and FIM theory. More importantly, the problem is abstractly simplified into a mathematical model that is tractable for engineering;
2. The second aspect is to consider the problems of the mayfly algorithm with poor global search ability, small population diversity, and weak adaptive ability; the multi-strategy fusion improved adaptive mayfly algorithm (MIAMA) is proposed based on a reverse learning mechanism, chaotic mapping, and nonlinear inertia weight factors;
3. The last aspect involves applying the proposed method to the system model of the bearing-only sensor network and verifying the effectiveness and superiority of this solution for the optimal detection array layout problem through simulation experiments.

The remainder of this paper is organized as follows: Section 2 constructs a system model for locating and tracking the target through angle of arrival (AOA) via a bearing-only sensor network and performs the systematic observability analysis. In Section 3, by deriving the FIM and CRLB of the system model, the optimal detection geometry configuration in the bearing-only sensor network is analyzed. Section 4 introduces the MIAMA, which optimizes and solves the problem based on the objective function of geometric accuracy dilution. Section 5 presents the verification of the effectiveness and superiority of the proposed MIAMA through several sets of simulation experiments. Finally, the research content and future work directions of this paper are summarized.

Notation: This paper employs bold letters to signify vectors or matrices. We define $\gamma_{i}\in \left(-\pi ,\pi \right]$. tr(·) and det(·) as the trace and the determinant of the matrix enclosed within the brackets, respectively. $diag\left[\cdot \right]$ denotes the diagonal matrix with the elements of a vector as diagonal elements. ${\left(\right)}^{T}$ and ${\left(\right)}^{-1}$ represent the transpose and inverse of a matrix, respectively. E(·) denotes expectation.

## 2. System Model

In the observation model of the bearing-only sensor network, each sensor can independently measure the elevation and azimuth angles to the target, and two sensor nodes and the target form a triangular geometric relationship for tracking and localization. The target position is determined by the intersection point of the lines of sight (LOS) from the sensor node to the aiming target [21]. This method of target tracking and positioning is based on the principle of AOA measurement.

The communication topology of the bearing-only sensor network is an undirected connected network G(N,R). $N=\left\{1,2,\cdots ,N\right\}$ is the set of sensor nodes. R denotes the set of connections between nodes. An edge (i,j)∈R indicates that node *i* can receive information from node *j*. For each node i∈N, if node *j* is included in its neighbors, $N_{i}=\left\{j\left|(i,j)\in R\right.\right\}$, otherwise $N_{i}\setminus \left\{j\right\}$ [22].

**Figure 1:**
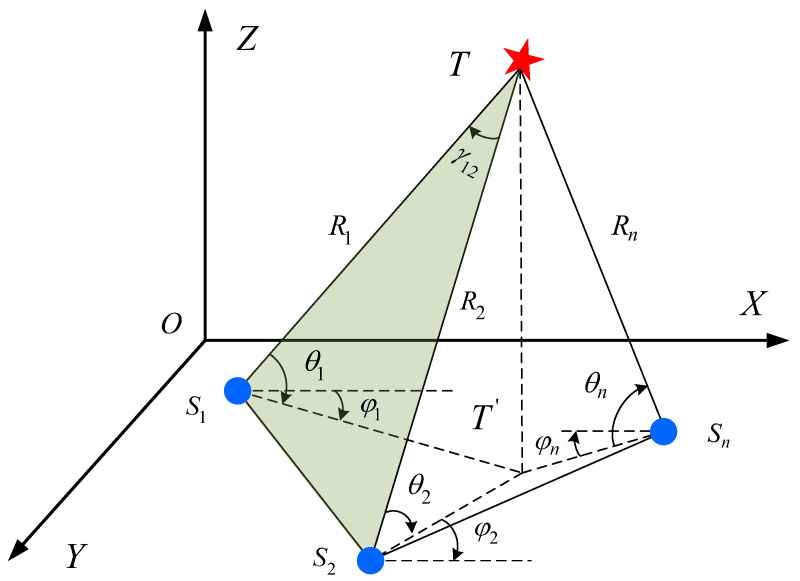
Bearing-only detection model.

In a 3D coordinate system, the geometric relationship between the bearing-only sensors and the target is shown in Figure 1. The elevation angle and azimuth angle measured by bearing-only sensor $S_{i}$ are $\theta_{i}$ and $\varphi_{i}$. Similarly, the two angles measured by $S_{j}$ are $\theta_{j}$ and $\varphi_{j}$. The coordinates of target *T* are ${\left(x_{t},y_{t},z_{t}\right)}^{T}$.

The vectors of the bearing-only sensor node pair and the target are $\vec{OS_{i}}={\left(x_{i},y_{i},z_{i}\right)}^{T}$, $\vec{OS_{j}}={\left(x_{j},y_{j},z_{j}\right)}^{T}$, and $\vec{OS_{T}}={\left(x_{t},y_{t},z_{t}\right)}^{T}$. According to the elevation and azimuth angle measured by the bearing-only sensors $S_{i}$ and $S_{j}$, the position coordinates of the target can be obtained through the triangulation positioning method.

(1) $$\left\{\begin{array}{c} x_{t}=x_{i}+\frac{\left(y_{j}-y_{i}\right)\cos\varphi_{j}\cos\varphi_{i}-\left(x_{j}-x_{i}\right)\sin\varphi_{j}\cos\varphi_{i}}{\sin(\varphi_{i}-\varphi_{j})}\\ y_{t}=y_{i}+\frac{\left(y_{j}-y_{i}\right)\cos\varphi_{j}\sin\varphi_{i}-\left(x_{j}-x_{i}\right)\sin\varphi_{j}\sin\varphi_{i}}{\sin(\varphi_{i}-\varphi_{j})}\\ z_{t}=z_{i}+\frac{\left(y_{j}-y_{i}\right)\cos\varphi_{j}\sin\theta_{i}-\left(x_{j}-x_{i}\right)\sin\varphi_{j}\sin\theta_{i}}{\cos\theta_{i}\sin\left(\varphi_{i}-\varphi_{j}\right)} \end{array}\right.$$

When $\theta_{i}=\theta_{j}$ and $\varphi_{i}=\varphi_{j}$, the target line-of-sight of the sensor node pair coincides, and the system of equations has no solution.

**Figure 2:**
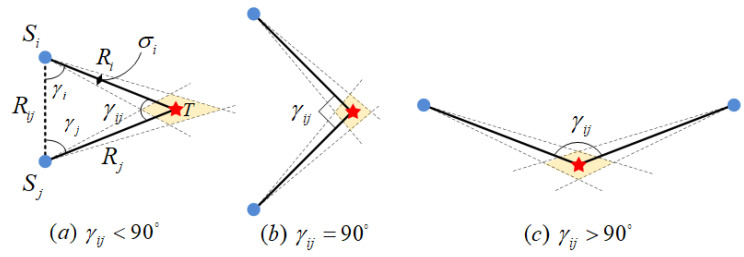
Geometry of the bearing-only sensor node.

In the observation model of the bearing-only sensor network, two sensor nodes and the target form a triangular geometric relationship for state estimation, as shown in Figure 2. The distance from $S_{i}$ to *T* is $R_{i}$, and the distance from $S_{j}$ to *T* is $R_{j}$. $R_{ij}$ represents the distance between $S_{i}$ and $S_{j}$. $\gamma_{ij}$ represents the line-of-sight (LOS) separation angle between Si and Sj. $\gamma_{i}$ is the angle between $S_{i}$ and *T*. Similarly, the meaning of $\gamma_{j}$ can be obtained.

Analyzing intuitively from the perspective of space geometry, the three typical geometric configurations are the $\gamma_{ij}<90^{{}^{\circ}}$, $\gamma_{ij}=90^{{}^{\circ}}$, and $\gamma_{ij}>90^{{}^{\circ}}$ scenarios. The propagation of the angular measurement error of the bearing-only sensor through the geometric structure will form a probability area surrounding the positioning error around the target. The true coordinates of the target may exist anywhere within this overlapping area. Obviously, when the probability area of the positioning error is larger, it indicates that the positioning accuracy of the system is smaller.

On the basis of spatial geometric analysis, the problem is numerically analyzed. When a pair of pure orientation sensor nodes detects a target, three spatial coordinates determine a plane, which is defined as the observation plane. In the positioning triangle $\Delta S_{i}TS_{j}$ of this plane, there is

(2) $$\left\{\begin{array}{cc} R_{i} & =\frac{\sin\gamma_{j}}{\sin\gamma_{ij}}R_{ij}\\ R_{j} & =\frac{\sin\gamma_{i}}{\sin\gamma_{ij}}R_{ij}\\ \gamma_{ij} & =\pi -(\gamma_{i}+\gamma_{j}) \end{array}\right.$$

The partial derivative of the distance $R_{i}$ of sensor node *i* is expressed as

(3) $$\begin{array}{cc} \delta R_{i} & =\frac{\sin\gamma_{j}}{\sin\gamma_{ij}}\delta R_{ij}+\frac{R_{ij}\cos\gamma_{j}}{\sin\gamma_{ij}}\delta \gamma_{j}-\frac{R_{ij}\cos\gamma_{ij}\sin\gamma_{j}}{\sin^{2}\gamma_{ij}}\delta \gamma_{ij}\\ & =\frac{1}{\sin\gamma_{ij}}\left\{\sin\gamma_{j}\delta R_{ij}-R_{ij}\sin\gamma_{j}\cot\left(\gamma_{i}+\gamma_{j}\right)\delta \gamma_{i}+R_{ij}\left[\cos\gamma_{j}-\sin\gamma_{j}\cot\left(\gamma_{i}+\gamma_{j}\right)\right]\delta \gamma_{j}\right\} \end{array}$$

According to the analysis results, the error in the estimation of $S_{i}$ for the distance is related to $\delta R_{ij}$, $\delta \gamma_{i},$ and $\delta \gamma_{ij}$. When the LOS separation angle $\gamma_{ij}=\pi /2$, there is the smallest error in distance estimation. When the LOS separation angle reaches 0 or π, the distance estimation error reaches ∞.

At this moment, the sensor nodes are aligned in a straight line, the observation model degenerates into a single-sensor detection problem, the target observability is reduced, and the distance estimation cannot be achieved [23]. This is consistent with the conclusion of the previous Equation (1) analysis.

Therefore, in order to ensure that the bearing-only sensor can conclude the estimation process for the target, the triangular geometric relationship between the sensor node pair and the target must be satisfied. The quantity of nodes in the sensor network is N,(N≥2). When the LOS separation angle is closer to $\gamma_{ij}=\pi /2$, the distance estimation effect is better.

## 3. Analysis of Optimal Detection Array

Different array layouts in bearing-only sensor networks can significantly impact detection performance [24]. Therefore, to address the issue of optimal detection array planning, it is necessary to analyze the positioning theory of the bearing-only sensor network.

### 3.1. CRLB and FIM

In this paper of a constructed system model of a bearing-only sensor network, the measurement of sensor *i* is

(4) $${\hat{m}}_{i}\left(T\right)=m_{i}\left(T\right)+e_{i}={\left[\theta_{i}\left(T\right),\varphi_{i}\left(T\right)\right]}^{T}+{\left[e_{\theta i},e_{\varphi i}\right]}^{T}$$

where $e_{\theta i}$ and $e_{\varphi i}$ are components of the measurement error vector $e_{i}$.

(5) $$\left\{\begin{array}{c} \theta_{i}\left(T\right)=\arctan\frac{x_{t}-x_{si}}{y_{t}-y_{si}}\\ \varphi_{i}\left(T\right)=\arctan\frac{z_{t}-z_{si}}{\sqrt{{\left(x_{t}-x_{si}\right)}^{2}+{\left(y_{t}-y_{si}\right)}^{2}}} \end{array}\right.$$

The set of measurement values of *N* sensors is

(6) $$\begin{array}{cc} \hat{M} & =M\left(T\right)+e={\left[m_{1}^{T},m_{2}^{T},\cdots ,m_{N}^{T}\right]}^{T}+e={\left[e_{1}^{T},e_{2}^{T},\cdots ,e_{N}^{T}\right]}^{T} \end{array}$$

Utilize R to symbolize the covariance matrix of $e_{i}$.

(7) $$R=\mathrm{diag}{\left[R_{i}\right]}_{2N\times 2N}$$

where $R_{i}=\mathrm{diag}\left(\sigma_{\theta }^{2},\sigma_{\varphi }^{2}\right)$.

For an unknown state parameter T, CRLB is the minimum variance achievable by an unbiased estimator $\hat{T}$ under regularity conditions, and it is equivalent to the inverse of the FIM [25]. FIM is used to evaluate the uncertainty of position estimates. In the bearing-only sensor network, the measurements are the AOAs of the target relative to each sensor. The elements of FIM provide covariance information of the position coordinates to describe the uncertainty of the parameter estimates [26].

The Cramer–Rao inequality is

(8) $$E\left[\left(\hat{T}-T\right){\left(\hat{T}-T\right)}^{T}\right]\geq F^{-1}\left(T\right)≜CRLB$$

where F(T) is FIM. In general, if F(T) is non-singular, a partial estimator of ***T*** with finite variance exists theoretically. FIM quantifies the amount of information about ***T*** carried by the measurement set. The detF(T) is inversely proportional to the uncertainty area of ***T***.

For a measurement set $\hat{M}$ of the network system model, FIM is

(9) $$F\left(T\right)=\nabla_{T}M{\left(T\right)}^{T}R^{-1}\nabla_{T}M\left(T\right)$$

When N=1, FIM can be expressed as:

(10) $$F\left(T\right)=\left[\begin{array}{ccc} \frac{\cos^{2}\beta_{1}}{\sigma_{\beta }^{2}r_{1}^{2}\cos^{2}\varphi_{1}}+\frac{\sin^{2}\beta_{1}\sin^{2}\varphi_{1}}{\sigma_{\varphi }^{2}r_{1}^{2}} & -\frac{\sin\beta_{1}\cos\beta_{1}}{\sigma_{\beta }^{2}r_{1}^{2}\cos^{2}\varphi_{1}}+\frac{\sin\beta_{1}\cos\beta_{1}\sin^{2}\varphi_{1}}{\sigma_{\varphi }^{2}r_{1}^{2}} & -\frac{\sin\beta_{1}\sin\varphi_{1}\cos\varphi_{1}}{\sigma_{\varphi }^{2}r_{1}^{2}}\\ -\frac{\sin\beta_{1}\cos\beta_{1}}{\sigma_{\beta }^{2}r_{1}^{2}\cos^{2}\varphi_{1}}+\frac{\sin\beta_{1}\cos\beta_{1}\sin^{2}\varphi_{1}}{\sigma_{\varphi }^{2}r_{1}^{2}} & \frac{\sin^{2}\beta_{1}}{\sigma_{\beta }^{2}r_{1}^{2}\cos^{2}\varphi_{1}}+\frac{\cos^{2}\beta_{1}\sin^{2}\varphi_{1}}{\sigma_{\varphi }^{2}r_{1}^{2}} & -\frac{\cos\beta_{1}\sin\varphi_{1}\cos\varphi_{1}}{\sigma_{\varphi }^{2}r_{1}^{2}}\\ -\frac{\sin\beta_{1}\sin\varphi_{1}\cos\varphi_{1}}{\sigma_{\varphi }^{2}r_{1}^{2}} & -\frac{\cos\beta_{1}\sin\varphi_{1}\cos\varphi_{1}}{\sigma_{\varphi }^{2}r_{1}^{2}} & \frac{\cos^{2}\varphi_{1}}{\sigma_{\varphi }^{2}r_{1}^{2}} \end{array}\right]$$

When detF(T)=0, there is no unbiased estimator for T. By analogy, when N≥2, we can obtain ∀i,j∈{1,2,⋯,N}, $r_{i}=∥r_{i}∥$ is a scalar, we have $r_{i}=∥s_{i}-T∥=∥{s^{\prime }}_{i}-T∥$, and the bearing-only sensor *i* moving from $s_{i}\left(x_{si},y_{si},z_{si}\right)$ to ${s^{\prime }}_{i}\left(2x_{T}-\right.\left.x_{si},2y_{T}-y_{si},2z_{T}-z_{si}\right)$ will not change the value of the FIM determinant, which means that there may be multiple optimal geometric configurations. Next is the analysis of space geometry based on the non-unique theory of optimal geometric configuration.

### 3.2. Optimal Geometric Array

In the bearing-only sensor network studied in this paper, CRLB is calculated by considering the variance of the node pair measurements and the true position of *T*. In the process of system state estimation, the more reasonable the sensor target geometry has a larger detF(T), the smaller the uncertainty range of target estimation. More importantly, detF(T) with different geometric formations may have the same CRLB, and the optimal geometric configuration may not be unique.

In the scenario where *N* bearing-only sensors track a single target *T*, the error in measuring angle is σ [27]. The analysis of the optimal detection array is as follows.

**Figure 3:**
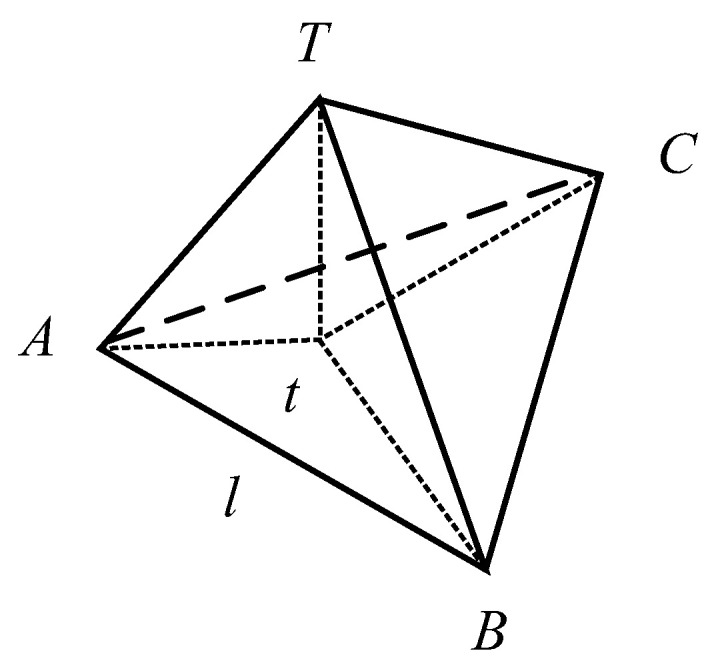
(*N* = 3) Optimal detection geometry under constraints.

**Figure 4:**
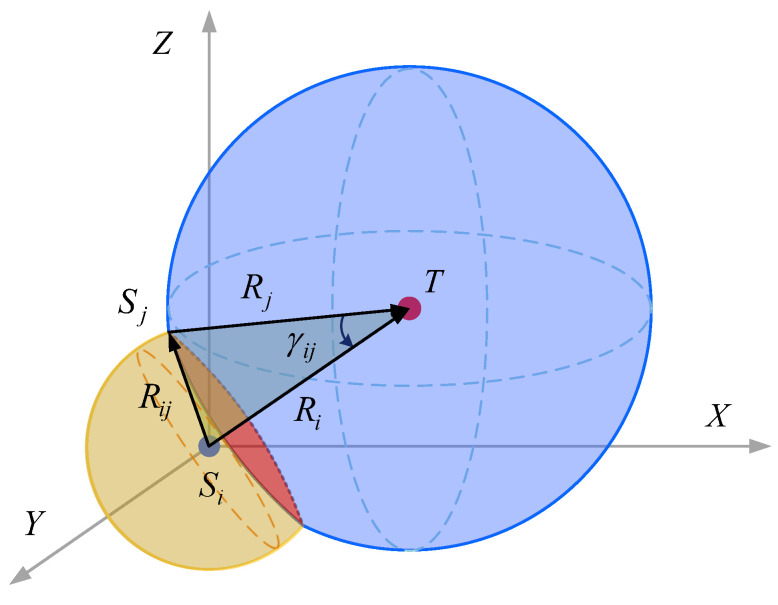
Geometric analysis of the optimal detection array.

1. When N=1, there is no unbiased estimator, no triangulation model is formed, and the target distance cannot be estimated.
2. When N=2, $R_{1}$ and $R_{2}$ are fixed if and only when $\gamma_{12}=\pi /2$, it is the optimal detection geometric configuration, and when $R_{i}\to \infty$ or the two bearing-only sensors are collinear with *T*, and the system cannot obtain the estimated result.
3. When N=3, det(F(T)) can be obtained by simplifying.
  (11) $$\begin{array}{ccc} \det(F(T)) & =\sum_{1\leq i<j\leq 3}\frac{R_{i}^{2}+R_{j}^{2}}{\sigma^{6}R_{i}^{4}R_{j}^{4}}\left(1-\cos^{2}\gamma_{ij}\right) & +\frac{2}{\sigma^{6}R_{1}^{2}R_{2}^{2}R_{3}^{2}}\cdot \left(1-\cos\gamma_{12}\cos\gamma_{13}\cos\gamma_{23}\right) \end{array}$$
  The optimal geometry of the three sensor systems for collaboratively detecting a single target appears at $\gamma_{12}=\gamma_{23}=\gamma_{13}=\pi /2$. Position three sensors at the vertices of an equilateral triangle with side length *l* under the constraint of fixed distance between adjacent sensors. The distance from *T* to the center of mass is $l/\sqrt{6}$, as shown in Figure 3.
  It is assumed that the center of mass of an equilateral triangle of size *l* is *t*, and the three bearing-only sensors are located at vertices A, B, and C, respectively [27].
4. When N≥4, the optimal detection array is not unique. Therefore, when the distance from each bearing-only sensor to the target is fixed, ∀i,j,k∈{1,2,⋯,N}, the maximizing detection efficiency is
  (12) $$F\left(\gamma \right)=\min\sum_{\Omega 1}\cos^{2}\gamma_{ij}+\sum_{\Omega 2}\cos\gamma_{ij}\cos\gamma_{ik}\cos\gamma_{jk}$$

where Ω1={(i,j)∣1≤i<j≤N}, Ω2={(i,j,k)∣1≤i<j<k≤N}, $\left|\Omega 2\right|=C_{N}^{3}$. The problem is simplified to how to reasonably arrange *N* bearing-only sensors on the circle where two balls intersect [27], as shown in Figure 4.

In the three-dimensional space, the spatial position of the first node $S_{i}$ in the sensor network is known, with the coordinates of node $S_{i}$ as the center of the sphere, the fixed adjacent communication distance as the radius, and the formed sphere is represented as $Q_{i}$. Then, take the coordinates of target *T* as the center and the distance from node $S_{i}$ to target *T* as the radius, and the formed ball is represented as $Q_{t}$. Sphere $Q_{i}$ and $Q_{t}$ intersect at circle ${⊙}_{i}$. Now the spatial position of the second node $S_{j}$ in the sensor network can be determined to form the optimal detection geometry with node $S_{i}$. But in fact, every point on the circle ${⊙}_{i}$ can be used as the spatial position of node $S_{j}$, thus forming an infinite point set $\Phi_{j}$, which makes subsequent models unable to perform effective calculations. From the longitudinal plane and the normal plane passing through the center of the circle, four points in $\Phi_{j}$ can be determined as the coordinates of the second node $S_{j}$. In the same way, use the coordinates of the second node $S_{j}$ as the center of the sphere to find the third node $S_{k}$. By analogy, the geometric array of a bearing-only sensor network with a given number of nodes can be designed in sequence.

The formation planning problem of a bearing-only sensor network is a multi-objective optimization problem; the function cannot obtain an analytical form due to its high nonlinearity [27]. Intelligent optimization algorithms can meet specific performance indicators to the greatest extent and automatically find the best solution in the search space to minimize or maximize the objective function. Numerical analytical methods are difficult in obtaining optimal solutions to complex nonlinear problems. Therefore, this paper chooses to use intelligent optimization algorithms to solve the optimal geometric formation of a bearing-only sensor network.

At the same time, consider that the target positioning accuracy is not only related to the observation array, but also depends on the errors of position and angle measurements for a bearing-only sensor itself. The GDOP indicator originates from these two errors of the sensor and propagates the error through relative geometric relationships, which can best describe the changes in target positioning capabilities that this paper focuses on. Therefore, the objective function of the proposed method is to select the GDOP indicator for iterative optimization.

## 4. The Proposed MIAMA

Based on the constructed system model and geometric array analysis, this paper proposes a multi-strategy fusion improved adaptive mayfly algorithm (MIAMA) to solve the optimization problem of geometric arrays in a bearing-only sensor network.

In the classic MA, each solution is a mayfly constantly flying in space, and the flight direction is the dynamic interaction of individual and social flight experiences. Each male mayfly will adjust its flight direction based on its own or companionable experience, which represents the global search and optimization ability. The female mayfly moves towards a mate that is better than itself. If the mate is weaker than itself, it will search for itself. Mayflies are ranked according to their fitness values and mate with each other to produce better offspring [28].

The reason why MA is chosen to solve this problem is mainly due to its excellent positive-feedback characteristics. This feature can quickly expand the initial difference and guide the entire system to evolve toward the optimal solution. Not only that, but the pairing mechanism of mayflies in the flight optimization algorithm is similar to the characteristic that bearing-only sensors can only be observed in pairs and is very suitable for solving the optimization problem of geometric arrays in a bearing-only sensor network.

However, traditional MA also has limitations that cannot be ignored, which are mainly reflected in three aspects. The first aspect is that the initial population diversity is small, and male and female mayfly individuals are derived from random distribution and are difficult to be evenly distributed in the state space. The second aspect is that the search has poor adaptability. In a situation involving a limited number of sensor nodes, MA cannot adaptively adjust the local search capability. The third aspect is that in scenarios with an extensive number of sensors, the global search capabilities of the MA may show shortcomings and need to be strengthened to a certain extent to deal with them. Based on the considerations of all the above factors, in order to enhance the population diversity, adaptability, and global search capability of MA, the MIAMA is proposed. The specific improvement measures are as follows:

Firstly, Tent chaos mapping was used to initialize the mayfly population. It enables the population to be uniformly distributed in the solution space to avoid the population being too concentrated or dispersed. In addition, it improves the diversity of the population, thereby enhancing the ability to escape from local optima.

Secondly, the adaptive inertia weight factor is introduced to achieve a more effective balance between global search and local development capabilities through adaptive dynamic adjustment, thereby improving the convergence accuracy of the algorithm.

**Figure 5:**
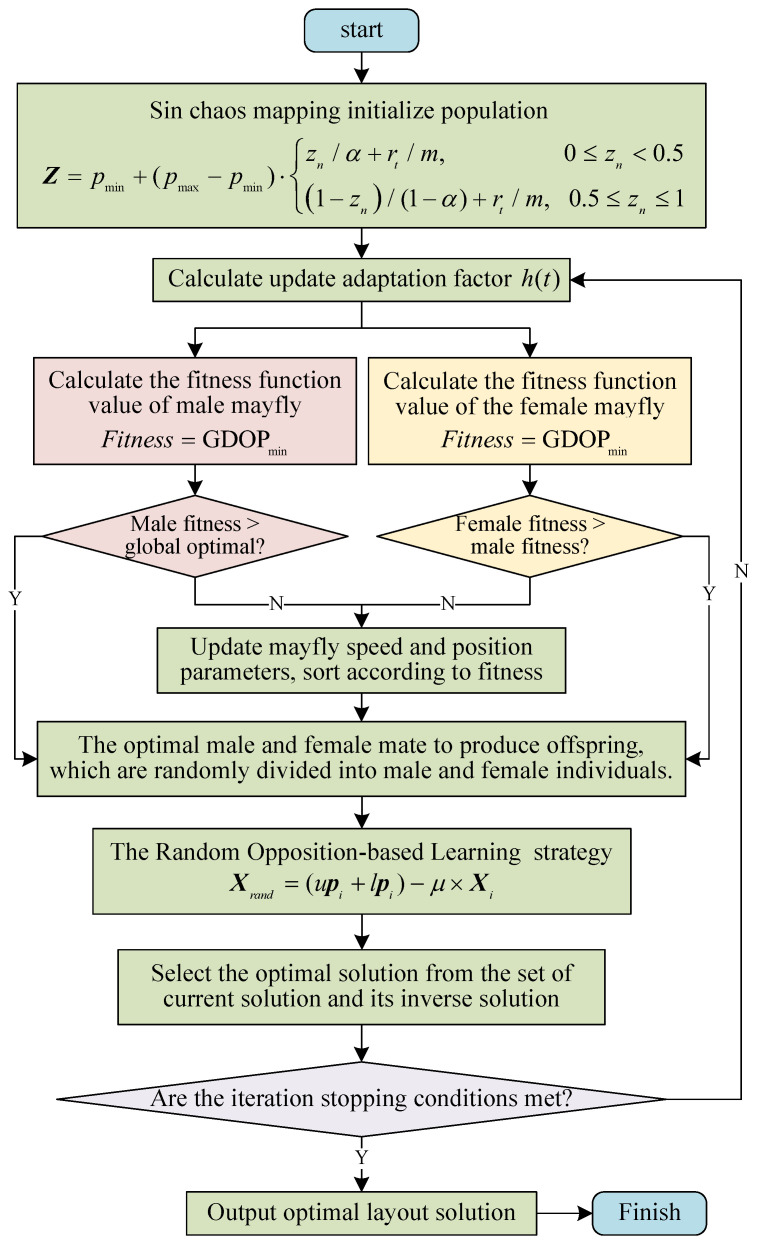
The flowchart of the mayfly optimization algorithm.

Finally, Random Opposition-based Learning (ROBL) was adopted to enhance the ability for global search, the stability, and the convergence speed. The flowchart of a bearing-only sensor network layout based on the proposed MIAMA is shown in Figure 5.

This paper adopts corresponding improvement strategies to address the various limitations of the algorithm one by one, and then integrates the three improvement strategies together in a rational manner to comprehensively enhance the performance of the algorithm. Importantly, we ensure that the introduced improvement strategies are decoupled and do not interfere with each other. Specific steps of the proposed MIAMA are as follows:

(1) Tent chaos mapping initialize population

An initialization population with a uniform distribution can effectively broaden the search range of the algorithm, thereby enhancing both convergence speed and solution accuracy. Random Tent chaos mapping has the characteristics of randomness, ergodicity, and regularity and is often used to optimize search problems to maintain population diversity and jump out of local optimal solutions. The Random Tent chaos mapping sequence is expressed as

(13) $$z_{n+1}=\left\{\begin{array}{cc} z_{n}/\alpha +r_{t}/m, & 0\leq z_{n}<0.5\\ \left(1-z_{n}\right)/\left(1-\alpha \right)+r_{t}/m, & 0.5\leq z_{n}\leq 1 \end{array}\right.$$

where $r_{t}$ is a random number in $\left(0,1\right)$, which affects the degree of chaos in two aspects. Firstly, the equation introduces randomness into the system, ensuring that the evolution of the system is not completely deterministic. This means that even with the same initial conditions, the system may evolve differently over time due to different random values of $r_{t}$. In addition, there is sensitivity to initial conditions, where even small differences in initial conditions can lead to dramatically different outcomes over time. The random term $r_{t}$ amplifies this sensitivity by introducing unpredictable changes into the system. *m* is the number of elements in the Tent chaos sequence. α is a random perturbation uniformly distributed in the range $\left(-1,1\right)$. After generating the chaotic sequence, it is mapped to the solution space Z.

(14) $$Z=p_{\min}+\left(p_{\max}-p_{\min}\right)\cdot z$$

where $p_{\max}$ and $p_{\min}$ are the upper and lower bounds of the solution space, respectively. Using the spatial coordinates of the bearing-only sensor network to generate Tent chaotic sequences according to Equation (13), we then map them to the solution space according to Equation (14) as the initial solutions of the mayfly population [29].

(2) Calculate fitness

This paper uses the Fitness to evaluate the quality of the sensor layout. In the sensor layout problem on the tangent circle, the minimization of the GDOP value was considered.

(15) $$Fitness=\mathrm{GDO}P_{\min}$$

where GDOP is related to the position vector $X(X\in R^{n})$, measurement error covariance matrix $R(R\in R^{n})$, and Jacobian matrix $H(H\in R^{m\times n})$. It is represented as $\mathrm{GDOP}=\sqrt{\mathrm{trace}[H^{-1}R{\left(H^{-1}\right)}^{T}]}$. *n* represents the sensor number indicator, and *m* is the number of sensors in the optimal layout of the current sensor network [30].

(3) Iterate

The inertia weight factor $h\left(t\right)$ plays the guiding role in the search and development capabilities of the algorithm, reflecting the ability of the mayfly to learn from certain prior behaviors. As h(t) increases, it enhances the global search capability. However, as h(t) decreases, it enhances the local search capability. This paper introduces a nonlinear decreasing adaptive gravity coefficient to better balance global search and local development capabilities.

(16) $$h\left(t\right)={\left(1-t/D\right)}^{\sqrt[{\rho }]{t/D}}\cdot \Gamma \left(\eta ,1-t/D\right)$$

where *t* is the current time step, *D* is the maximum number of iterations, and ρ=0.8 is the control coefficient of inertia weight obtained from experience. Γ(η,1−t/D) is an incomplete gamma function. η is a random variable greater than zero but less than ρ.

For each iteration, the individual in the state space is updated through the flight strategy of the MIAMA, the fitness of the new position is computed, and then the merits of the sensor layout are evaluated according to the objective function. The location update of the mayfly is related to the neighborhood individuals.

(17) $$\left\{\begin{array}{c} x_{i}^{t+1}=x_{i}^{t}+v_{i}^{t+1}\\ y_{i}^{t+1}=y_{i}^{t}+v_{i}^{t+1} \end{array}\right.$$

where $x_{i}^{t+1}$ and $y_{i}^{t+1}$ represent the individual *i* at the tth iteration, respectively. The flight speed in the iteration is represented by $v_{i}^{t+1}$.

The update of velocity for a male mayfly is

(18) $$v_{ij}^{t+1}=\left\{\begin{array}{cc} h\left(t\right)\cdot v_{ij}^{t}+a_{1}e^{-\beta r_{B}^{2}}\left(\;B_{ij}-x_{ij}^{t}\right)+a_{2}e^{-\beta r_{A}^{2}}\left(\;A_{ij}-x_{ij}^{t}\right), & f\left(x_{i}^{t}\right)\leq f_{\min}\\ h\left(t\right)\cdot v_{ij}^{t}+d\cdot r, & f\left(x_{i}^{t}\right)\leq f_{\min} \end{array}\right.$$

where $v_{ij}^{t+1}$ is the speed of *i* in the jth dimension at step t+1. $a_{1}=1$, $a_{2}=1.5$ are both positive constants representing attraction forces, which are employed to measure the influence of the individual and the optimal individual on the current flight speed, respectively. $B_{ij}$ is the historical optimal position of individual *i*. $A_{ij}$ represents the optimal value among all individuals. β=2 represents the visibility coefficient of the mayfly, regulating the visible range. $r_{B}$ represents the distance between the historical optimal value and the current value. $r_{A}$ represents the distance between the optimal individual value and the current value [31].

(19) $$∥x_{i}-X_{i}∥=\sqrt{\sum_{j=1}^{n}{\left(x_{ij}-X_{ij}\right)}^{2}}$$

where $x_{ij}$ is the jth component of the mayfly *i*. $X_{i}$ represents the corresponding $B_{ij}$ and $A_{ij}$.

The update of velocity for a female mayfly is expressed as

(20) $$v_{ij}^{t+1}=\left\{\begin{array}{cc} h\left(t\right)\cdot v_{ij}^{t}+a_{2}e^{-\beta r_{c}^{2}}\left(x_{ij}^{t}-y_{ij}^{t}\right), & f\left(y_{i}\right)>f\left(x_{i}\right)\\ h\left(t\right)\cdot v_{ij}^{t}+k\cdot r, & f\left(y_{i}\right)\leq f\left(x_{i}\right) \end{array}\right.$$

where $r_{c}$ is the Cartesian distance between female individuals and male individuals, which can be obtained from Equation (19). Male mayflies consistently engage in a courtship dance above the water, and *d* is the courtship dance coefficient. *k* is the random walk coefficient. The female mayflies engage in random flight when they are not attracted by the male mayflies. *r* is a random number between $\left[-1,1\right]$ [32].

(4) Choose the optimal solution

Opposition-based Learning (OBL) is an effective optimization approach [33]. The Random Opposition-based Learning (ROBL) strategy is built upon this foundation. Firstly, the current solution is subtracted from the sum of the upper bound $up_{i}$ and lower bound $lp_{i}$ to generate a reverse solution. Then, a random number is introduced. Finally, the fitness function values of the current resolution and the inverse resolution are compared. We include the optimal one in the subsequent iteration. This is employed to strengthen the global search capability of MIAMA and reduce the likelihood of converging to a local optimum and getting stuck.

(21) $$X_{rand}=\left(up_{i}+lp_{i}\right)-\mu \times X_{i}$$

where $X_{rand}$ is a random reverse solution, and μ is a random number between 0 and 1.

The iteration stop condition is whether the MIAMA reaches the set number of iterations, and then among all iteration results, the individual with the highest fitness is the optimal calculation result.

## 5. Simulation Experiment

To validate the effectiveness and superiority of the proposed MIAMA, the three comparative experiments were set up through a Matlab simulation. The bearing-only sensor network tracks and locates the single target in 3D space. Assuming that all bearing-only sensors have uniformly completed time and space registration [34], the initial simulation parameters are shown in Table 1. $X_{t}$ = (500 m, 500 m, 500 m) is the initial state of the target. $S_{i}$ corresponds to the spatial position of the ith node in the bearing-only sensor network.

**Figure 6:**
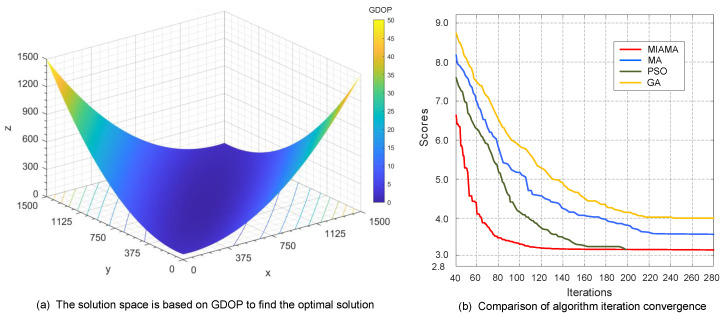
Results and comparison of the first set of simulation experiments.

In the first set of simulation scenarios, a system network of six bearing-only sensor nodes was set up to track and locate the target. Through MIAMA calculations, the optimal detection array of the bearing-only sensor network was as follows: $S_{1}$ ($1.00\times 10^{3}$$1.00\times 10^{3}$$1.00\times 10^{3}$), $S_{2}$ ($9.75\times 10^{2}$$9.52\times 10^{2}$$1.03\times 10^{3}$), $S_{3}$ ($9.75\times 10^{2}$$9.52\times 10^{2}$$1.07\times 10^{3}$), $S_{4}$ ($9.36\times 10^{2}$$1.02\times 10^{3}$$1.10\times 10^{3}$), $S_{5}$ ($9.05\times 10^{2}$$1.05\times 10^{3}$$1.07\times 10^{3}$), $S_{6}$ ($9.05\times 10^{2}$$1.05\times 10^{3}$$1.03\times 10^{3}$). The process of MIAMA searching for the optimal solution based on GDOP in the solution space is shown in Figure 6a. As the simulation time increases, the value of the GDOP objective function of algorithm gradually decreases, and the function decreases faster and in a clearer direction. It is proved that the MIAMA can effectively solve the optimal observation geometric formation.

The iteration curves of the proposed MIAMA with traditional MA, PSO, and GA are compared and analyzed, as shown in Figure 6b. MIAMA can converge to the optimal solution in 127 generations, which is superior to other algorithms. MA, PSO, and GA terminate the optimization process at generations 223, 196, and 242, respectively. MIAMA not only has the fastest downward trend, but also has a higher system state estimation accuracy. The superiority of the algorithm and its optimal objective function value are ranked as MIAMA(3.15) > PSO(3.15) > MA(3.66) > GA(3.98).

**Figure 7:**
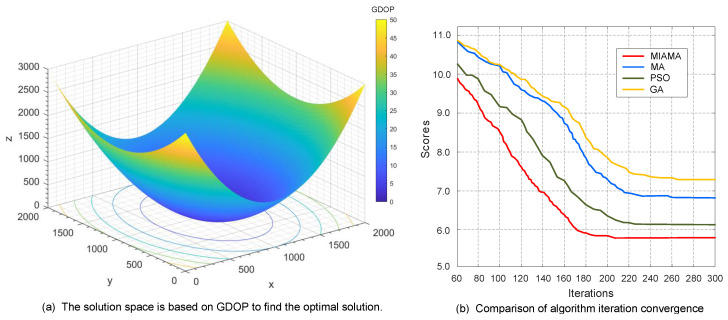
Results and comparison of the second set of simulation experiments.

In the second set of simulation scenarios, a system network of eight bearing-only sensor nodes was set up to track and locate the target. Through MIAMA calculations, the optimal detection array of the bearing-only sensor network was as follows: $S_{1}$ ($1.00\times 10^{3}$$1.04\times 10^{3}$$1.00\times 10^{3}$), $S_{2}$ ($1.00\times 10^{3}$$9.65\times 10^{2}$$1.00\times 10^{3}$), $S_{3}$ ($9.75\times 10^{2}$$9.52\times 10^{2}$$1.03\times 10^{3}$), $S_{4}$ ($9.32\times 10^{2}$$9.75\times 10^{2}$$1.07\times 10^{3}$), $S_{5}$ ($9.02\times 10^{2}$$1.01\times 10^{3}$$1.12\times 10^{3}$), $S_{6}$ ($9.04\times 10^{2}$$1.04\times 10^{3}$$1.10\times 10^{3}$), $S_{7}$ ($9.32\times 10^{2}$$1.07\times 10^{3}$$1.07\times 10^{3}$), $S_{8}$ ($9.71\times 10^{2}$$1.07\times 10^{3}$$1.03\times 10^{3}$). The process of MIAMA finding the optimal solution based on GDOP in the solution space is shown in Figure 7a. The GDOP objective function value of MIAMA also decreases progressively with simulation time, but the direction of descent for the function becomes anisotropic. The system operation is more complex compared to scenarios with small-scale nodes, but it can still approach the optimal solution set in a stable direction.

On this basis, the iteration curves of the four algorithms MIAMA, MA, PSO, and GA are also compared and analyzed, as shown in Figure 7b. MIAMA can converge to the optimal solution in 207 generations, which is significantly better than other algorithms. Among them, MA, PSO, and GA terminate the optimization process at generations 260, 226, and 264, respectively. MIAMA not only has the fastest downward trend, but also has a higher system state estimation accuracy. The superiority of the algorithm and its optimal objective function value are ranked as MIAMA(5.72) > PSO(6.24) > MA(6.78) > GA(7.42).

**Figure 8:**
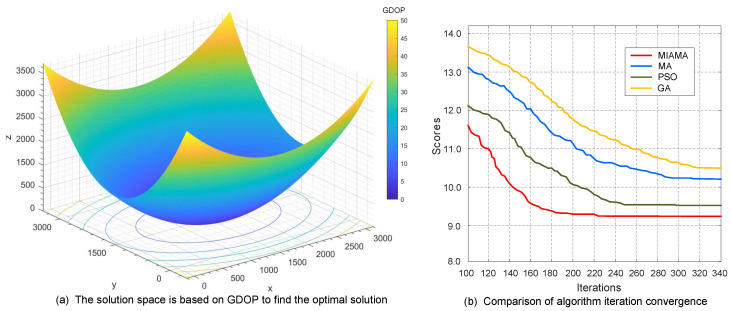
Results and comparison of the third set of simulation experiments.

In the third set of simulation scenarios, a system network of ten bearing-only sensor nodes was set up to track and locate the target. Through MIAMA calculations, the optimal detection array of the bearing-only sensor network was as follows: $S_{1}$ ($1.00\times 10^{3}$$1.04\times 10^{3}$$1.00\times 10^{3}$), $S_{2}$ ($9.82\times 10^{2}$$9.72\times 10^{2}$$1.02\times 10^{3}$), $S_{3}$ ($9.55\times 10^{2}$$9.55\times 10^{2}$$1.05\times 10^{3}$), $S_{4}$ ($9.12\times 10^{2}$$9.52\times 10^{2}$$1.09\times 10^{3}$), $S_{5}$ ($8.83\times 10^{2}$$9.72\times 10^{2}$$1.12\times 10^{3}$), $S_{6}$ ($8.61\times 10^{2}$$1.01\times 10^{3}$$1.14\times 10^{3}$), $S_{7}$ ($8.82\times 10^{2}$$1.03\times 10^{3}$$1.12\times 10^{3}$), $S_{8}$ ($9.10\times 10^{2}$$1.05\times 10^{3}$$1.05\times 10^{3}$), $S_{9}$ ($9.52\times 10^{2}$$1.05\times 10^{3}$$1.05\times 10^{3}$), $S_{10}$ ($9.82\times 10^{2}$$1.03\times 10^{3}$$1.02\times 10^{3}$). The process of MIAMA finding the optimal solution based on GDOP in the solution space is shown in Figure 8a. The GDOP objective function value of MIAMA also gradually decreases with the simulation time. The decline of the function is similar to that of the second group. The system operation is relatively more complex, but it can still approach the optimal solution set with a stable direction and speed.

On this basis, the iteration curves of the four algorithms MIAMA, MA, PSO, and GA are also compared and analyzed, as shown in Figure 8b. MIAMA can converge to the optimal solution in 224 generations, which is significantly better than other algorithms. Among them, MA, PSO, and GA terminate the optimization process at generations 312, 296, and 319, respectively. MIAMA not only has the fastest downward trend, but also has a higher system state estimation accuracy. The superiority of the algorithm and its optimal objective function value are ranked as MIAMA(9.28) > PSO(9.62) > MA(10.25) > GA(10.66).

Through the simulation experiments of three groups of network nodes of different sizes, it is, firstly, verified that that the proposed MIAMA can reasonably and effectively solve the optimal geometric configuration problem for target tracking in a bearing-only sensor network. This is significantly reflected in the decreasing trend of the GDOP indicator in the state solution space. Furthermore, taking a comprehensive view, from the first simulation experiment to the third simulation experiment, the number of the bearing-only sensor nodes gradually increased. On one hand, the convergence speed of various algorithms for solving this problem gradually decreases. This characteristic is manifested in the changing trend of the iteration numbers for PSO, which is 196→226→296, for MA is 223→260→312, for GA is 242→264→319, and for MIAMA is 127→207→224. On the other hand, the fluctuation trend of the gap between the convergence results of PSO and MIAMA is 0→0.52→0.34. The fluctuation trend of the gap between the convergence results of MA and MIAMA is 0.51→1.06→0.97. The fluctuation trend of the gap between the convergence results of GA and MIAMA is 0.83→1.7→1.38.

The comparison of these methods was performed in three sets of simulation experiments with different numbers of nodes to avoid limitations where a certain method may only perform superiorly in specific scenarios. Simultaneously, in the comparison and analysis with the MA, PSO, and GA methods, the superiority of MIAMA in solving the problem was confirmed through quantifying the iteration count and optimizing the objective function value. MIAMA demonstrated the ability to achieve better target localization results with faster convergence speed.

In summary, the proposed MIAMA integrates the principle of the Tent chaos mapping, adaptive inertia weight factor, and the mechanism of the Random Opposition-based Learning to solve the limitations of the traditional MA and greatly improve the adaptability and solving capabilities of the algorithm. In the planning of the optimal detection geometric configuration for a bearing-only sensor network, it shows the advantages of high detection accuracy and fast convergence speed, and can best meet the requirements of the system model. This research can also make an important technical reference for the fields of path planning and target tracking of bearing-only sensors. It has necessary academic research significance and engineering application value.

## 6. Discussion

The emergence of stealth maneuvering targets not only changes the combat mode of modern warfare, but, more importantly, it breaks the original strategic balance, causing modern military tactics and strategic defense systems to face unprecedented challenges [35]. The passive detection solution for a stealth maneuvering target in a bearing-only sensor network has the irreplaceable advantages of long detection range, high concealment, and strong anti-interference ability.

In the preliminary work on how to detect a stealth target efficiently and stably, through investigation of the development status and theoretical analysis, we have identified that conducting thorough research on the optimal detection geometric array in bearing-only sensor networks is crucial and necessary. Here, this problem is abstracted into a mathematical model that is convenient for engineering applications, and the MIAMA method is designed and implemented to ideally solve this problem. Finally, through simulation experiment scenarios of different scales, the verification of the research results in this paper underscores its crucial reference significance for the passive detection technology of bearing-only sensors, and can provide theoretical and technical support for anti-stealth solutions. The shortcoming of this research work is that there are some a priori empirical parameters in the proposed algorithm. In future work, machine learning training should be considered to obtain more reasonable empirical parameters of the system. Future research should also consider testing and evaluating the proposed method using real bearing-only sensors.

## 7. Conclusions

In a bearing-only sensor network, detection arrays with different geometric configurations will profoundly affect the detection accuracy and stability of the system. This paper, firstly, constructs the system model for bearing-only sensor detection and analyzes the observability of the system. Subsequently, relying on the theories of FIM and CRLB, the optimal detection geometry formation was analyzed. More importantly, taking into account the three limitations of the traditional MA, a multi-strategy fusion improvement was carried out to obtain a MIAMA that is more suitable for the system model. In conclusion, several groups of comparative simulation experiments were designed to validate the effectiveness and superiority of the proposed MIAMA.

## Figures and Tables

**Table 1:** Initial simulation parameters for the experiments.

Group	Axis	$S_{1}$	$S_{2}$	$S_{3}$	$S_{4}$	$S_{5}$	$S_{6}$	$S_{7}$	$S_{8}$	$S_{9}$	$S_{10}$
1	$x_{0}\left(10^{2}m\right)$	9.85	9.65	9.25	9.25	9.25	9.25	-	-	-	-
	$y_{0}\left(10^{2}m\right)$	9.85	9.52	9.52	9.85	10.52	10.54	-	-	-	-
	$z_{0}\left(10^{2}m\right)$	9.85	10.31	10.75	11.25	10.78	10.36	-	-	-	-
2	$x_{0}\left(10^{2}m\right)$	10.23	10.21	9.71	9.25	8.95	8.95	9.25	9.71	-	-
	$y_{0}\left(10^{2}m\right)$	10.45	10.25	9.71	9.71	10.27	10.46	10.76	10.74	-	-
	$z_{0}\left(10^{2}m\right)$	10.22	10.25	10.35	10.74	11.23	11.26	10.77	10.36	-	-
3	$x_{0}\left(10^{2}m\right)$	9.95	9.83	9.55	9.14	8.82	8.60	8.82	9.14	9.55	9.83
	$y_{0}\left(10^{2}m\right)$	9.95	9.71	9.55	9.55	9.71	9.95	10.34	10.55	10.52	10.36
	$z_{0}\left(10^{2}m\right)$	9.95	10.28	10.54	10.92	11.26	11.43	11.25	10.56	10.55	10.28

## Data Availability

The data that support the findings of this study are available upon request from the authors.

## Abbreviations

The following abbreviations are used in this manuscript:

AOA	Angle of Arrival
FIM	Fisher Information Matrix
CRLB	Cramer–Rao Lower Bound
LOS	Lines of Sight
CEP	Circular Error Probable
3D	Three-Dimensional
GDOP	Geometric Dilution of Precision
MIAMA	Multi-strategy Fusion Improved Adaptive Mayfly Algorithm
MA	Mayfly Algorithm
PSO	Particle Swarm Optimization
GA	Genetic Algorithm
